# Laser Ablation for the Synthesis of Cu/Cu_2_O/CuO and Its Development as Photocatalytic Material for *Escherichia coli* Detoxification

**DOI:** 10.3390/ijms25136817

**Published:** 2024-06-21

**Authors:** Marcy Quintero, Marcela Manrique-Moreno, Henry Riascos, Ricardo A. Torres-Palma, Sandra Castro-Narvaez, Yenny P. Ávila-Torres

**Affiliations:** 1 Grupo de Investigación en Remediación Ambiental y Biocatálisis (GIRAB), Instituto de Química, Facultad de Ciencias Exactas y Naturales, Universidad de Antioquia UdeA, Calle 70 No. 52-21, A.A 1226, Medellín 050010, Colombia; marcy.quintero@udea.edu.co (M.Q.); ricardo.torres@udea.edu.co (R.A.T.-P.); 2 Grupo de Bioquímica Estructural de Macromoléculas, Instituto de Química, Facultad de Ciencias Exactas y Naturales, Universidad de Antioquia UdeA, Calle 70 No. 52-21, A.A 1226, Medellín 050010, Colombia; marcela.manrique@udea.edu.co; 3 Grupo de Investigación Plasma, láser y Aplicaciones, Departamento de Física, Facultad de Ciencias Básicas, Universidad Tecnológica de Pereira, Carrera 27 #10-02 Barrio Álamos, Pereira 660003, Colombia; hriascos@utp.edu.co; 4 Grupo de Investigación en Electroquímica y Medio Ambiente, Universidad Santiago de Cali, Calle 5 No. 62-00, Santiago de Cali 760035, Colombia

**Keywords:** reactive oxygen species, nanoparticles, mixed copper oxide, photocatalysis, disinfection activity, laser ablation

## Abstract

Advanced Oxidation Processes (AOPs) offer promising methods for disinfection by generating radical species like hydroxyl radicals, superoxide anion radicals, and hydroxy peroxyl, which can induce oxidative stress and deactivate bacterial cells. Photocatalysis, a subset of AOPs, activates a semiconductor using specific electromagnetic wavelengths. A novel material, Cu/Cu_2_O/CuO nanoparticles (NPs), was synthesized via a laser ablation protocol (using a 1064 nm wavelength laser with water as a solvent, with energy ranges of 25, 50, and 80 mJ for 10 min). The target was sintered from 100 °C to 800 °C at rates of 1.6, 1.1, and 1 °C/min. The composite phases of Cu, CuO, and Cu_2_O showed enhanced photocatalytic activity under visible-light excitation at 368 nm. The size of Cu/Cu_2_O/CuO NPs facilitates penetration into microorganisms, thereby improving the disinfection effect. This study contributes to synthesizing mixed copper oxides and exploring their activation as photocatalysts for cleaner surfaces. The electronic and electrochemical properties have potential applications in other fields, such as capacitor materials. The laser ablation method allowed for modification of the band gap absorption and enhancement of the catalytic properties in Cu/Cu_2_O/CuO NPs compared to precursors. The disinfection of *E. coli* with Cu/Cu_2_O/CuO systems serves as a case study demonstrating the methodology’s versatility for various applications, including disinfection against different microorganisms, both Gram-positive and Gram-negative.

## 1. Introduction

Infectious diseases caused by antibiotic-resistant microorganisms result in challenging treatment scenarios [1]. The rise of multi-drug-resistant (MDR) microorganisms stems from the misuse and overuse of antibiotics, which promotes the emergence of new resistance genes [2]. This has significant public health implications, especially with healthcare-associated infections (HAI) being a key concern [3,4].

Advanced Oxidation Processes (AOPs) have been widely studied for the elimination of microorganisms [5]. Photocatalysis, a specific type of AOP, involves the activation of a semiconductor by electromagnetic wavelengths. When irradiated, the semiconductor produces electron–hole pairs, resulting in the formation of reactive oxygen species (ROS). For example, photogenerated electrons can react with oxygen to form superoxide anion radicals, while holes in the valence band can interact with water to create hydroxyl radicals. These reactive species can damage bacterial cell membranes, oxidize proteins and polysaccharides, and alter cell permeability, ultimately causing cell deformation and leakage of cellular contents [6].

In this context, mixed copper oxides can exhibit different structures with and without electronic connection between the phases. A Cu/CuO/Cu_2_O heterojunction nanodisk has been utilized for the photodegradation of rhodamine dye. It has been observed that the energy of photoexcited electrons in combined phases of CuO and Cu_2_O can transform oxygen in superoxide radicals (^•^O_2_^−^), leading to superior photocatalytic activity under visible light excitation [7,8]. In the case of heterojunctions, the process of electronic transfer between these phases leads to improved absorption in the visible region, contributions from the copper plasmonic system to the conductivity, and reduced recombination. In this context, as shown in Table 1, photocatalytic applications of mixed copper oxides are very interesting for the degradation and removal of organic pollutants. However, there are two aspects to highlight. Only a few cases of their application in biological systems such as cancer cells, and even fewer in microorganism disinfection, have been reported. The second aspect concerns the preparation method, where chemical and electrochemical synthesis mainly govern, while processes such as laser ablation are absent. Additionally, although some studies investigated the reactive species responsible for the activities of the materials, most of them do not propose a concrete action mechanism, and the reports are mostly focused on structural aspects. Based on the above, this work presents a novel, fast, and clean synthesis of Cu/CuO/Cu_2_O nanoparticles and its application in bacterial disinfection.

In this work, we propose laser ablation as a method for synthesizing Cu/Cu_2_O/CuO NPs (Nanoparticles). It is easy to obtain multi-component oxides of the desired stoichiometric ratio using PLD (pulsed laser deposition). It has a high deposition rate, a short test period, and low substrate temperature requirements. The process is simple and flexible with great development potential and great compatibility. Process parameters can be arbitrarily adjusted, and there is no limit to the type of targets. We use a UV pulsed laser as the energy source for plasma generation as the high photon capability and high energy density are conditions that are non-polluting and easy to control. Cu/Cu_2_O/CuO was synthesized at a laser wavelength of 1064 nm using water as a solvent. The energy range was 25, 50, and 80 mJ over a period of 10 min. The target was sintered from 100–800 °C at 1.6, 1.1, and 1.0 °C/min [15].

Then, the morphological, optical, electrochemical, and photocatalytic properties of the antimicrobial Cu/Cu_2_O/CuO photocatalytic suspension were investigated. A photocatalytic mechanism was proposed to explain the reason for the enhanced photocatalytic degradation with the flexible Cu/Cu_2_O/CuO NPs.

## 2. Results

### 2.1. Optical Properties Characterization

**Figure 1:**
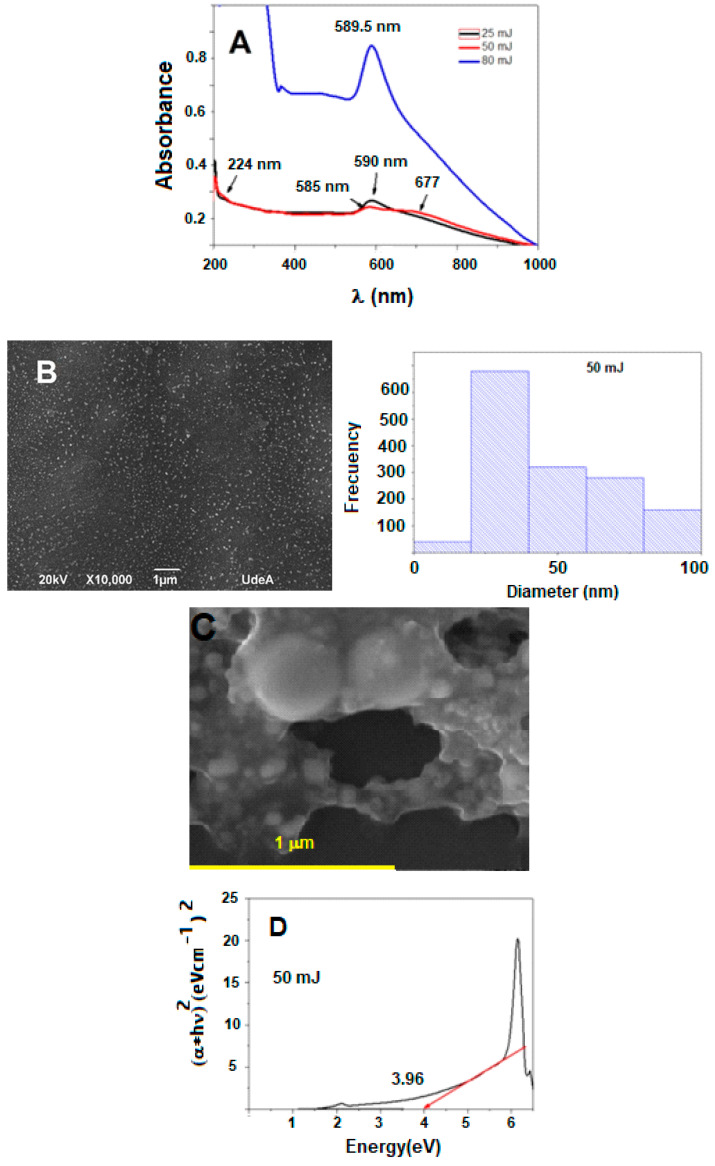
(**A**) Electronic spectra of NPs Cu/Cu_2_O/CuO with 25 mJ (black), 50 mJ (Red), and 80 mJ (blue), 10 min. (**B**) SEM for NPs Cu/Cu_2_O/Cu at 50 mJ, (**C**) SEM EDS for NPs Cu/Cu_2_O/CuO. (**D**) GAP estimation for NPs Cu/Cu_2_O/Cu at 50 mJ for electronic transition (black line) and GAP (red line).

The conditions of obtaining the NPs via laser ablation, such as deposition time, pulse energy, and laser wavelength, have an impact on the optical properties. Figure 1A shows the UV-VIS spectra of colloidal NPs at three different energies (25, 50, and 80 mJ) and ablation times (10 min). The spectra establish differences at 200 and 400 nm, which are characteristic bands of CuO and Cu_2_O absorption. The bands at 270 nm and 380 nm are assigned to CuO and Cu_2_O, respectively [16]. However, other authors indicate that the band near 210 nm corresponds to Cu_2_O and the band at 300 nm corresponds to CuO [17].

Figure 1B shows the SEM micrograph of one of those colloidal solutions (50 mJ), and its frequency as a function of the size is shown, while Figure 1C depicts TEM images where the spherical Cu/Cu_2_O/CuO NPs show an exposed surface in this analysis, as also seen in Table 2. Figure 1D shows the band GAP obtained to choose the wavelength for irradiation.

### 2.2. Electronic Properties Characterization

**Figure 2:**
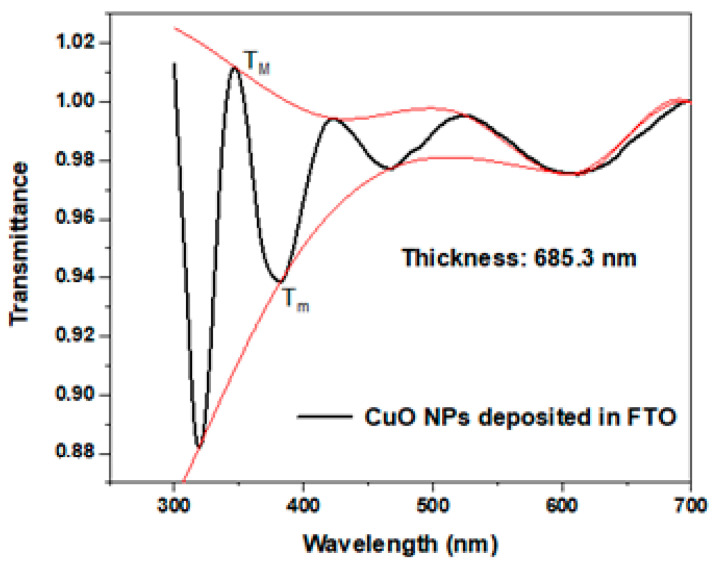
Thickness determination of Cu/Cu_2_O/CuO NPs on FTO by UV-VIs.

The thickness of Cu/Cu_2_O/CuO NP films on FTO (Fluorine-doped tin oxide electrodes) was determined by UV-Vis using Swanepoel’s method [18] (Figure 2). The control and time of the submersions in the deposition solution allow for obtaining dip-coating films with thicknesses of 685 ± 10 nm.

**Figure 3:**
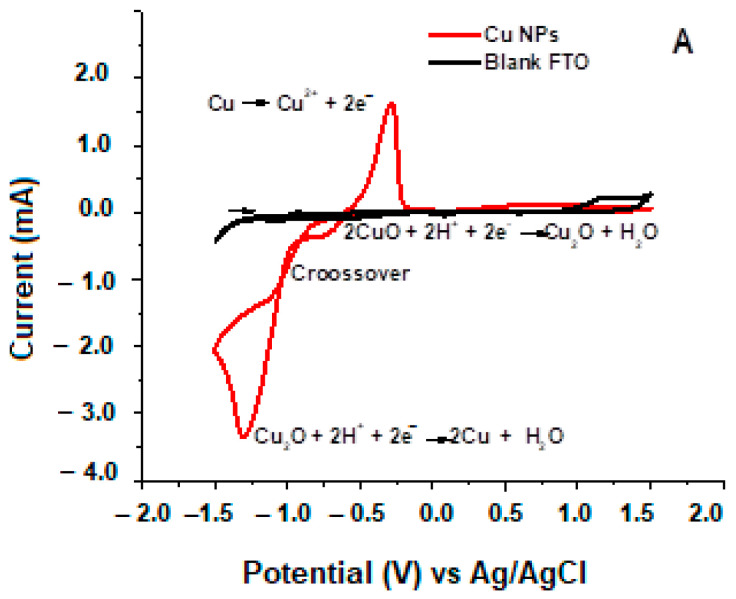

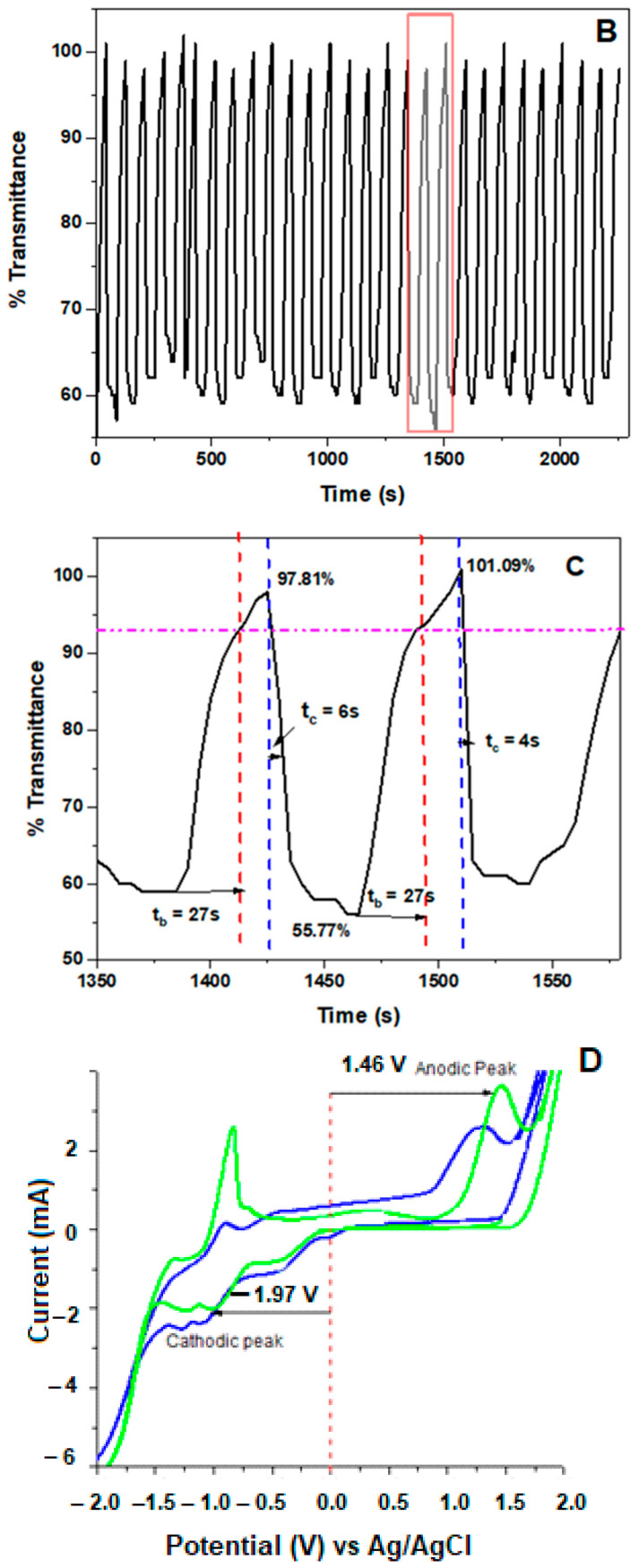

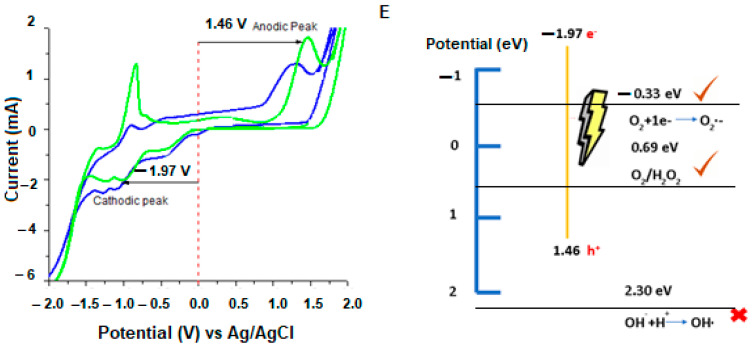
(**A**) Cyclic voltammetry response of Cu/Cu_2_O/CuO 50 mJ NPs deposited on FTO. (**B**) Charging and discharging in the light and dark regions: 0.1 M LiClO_4_ electrolyte, acidulated with HClO_4_ to pH 2.80. λ = 580 n, favored to 1500 s (**C**) Signal expansion of charging and discharging (red: initio transition, blue: finalization transition, purple: transition range) (**D**) Cyclic voltammetry response of NPs Cu/Cu_2_O/CuO with/without light in the presence of hydrogen peroxide. (**E**) Cathodic and anodic peaks and their potential correlated for the voltammogram in (**D**), X no generation of radical hydroxyl, 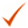 generation of superoxide anion radical.

The voltammograms of the NPs, as shown in Figure 3A, exhibit two peaks related to the reduction in copper species. The charge and discharge processes performed at 580 nm and potential changes between 1.5 V and −1.5 V, as shown in Figure 3B, show repeatability at 95% confidence (*p* = 0.013) of the electrical to optical conversion for 83 charge and discharge cycles. However, subsequent cycles evidenced losses of between 2 and 4% per 10 cycles. As an example, Figure 3C shows the enlargement of two cycles detailing how the bleaching time (tb), coloring time (tb), and contrast in the interconversion process are determined. The contrast was established by the difference between the highest and lowest percent transmittance. The tb corresponds to the duration from the time the film obtains the lowest percentage of transmittance until 93% of transmittance is reached and the signal generates a shoulder. The tc, on the other hand, corresponds to the time it takes for the film to change to 50% contrast. To understand the position of the valence and conduction band for Cu/Cu_2_O/CuO NPs, the voltammogram was run with and without light, showing the oxidative capabilities of the hole for H_2_O_2_ as a sacrificial species. Posteriorly, it is potentially electronically located in correlation with oxygen reactive species, hydroxyl radicals, and superoxide anion radicals, as shown in Figure 3D,E.

The spectroelectrochemical study of CuO NPs obtained by laser desorption and deposited dip coating on FTO can be seen in Table 3.

### 2.3. Antimicrobial Activity against E. coli ATCC 25922

**Figure 4:**
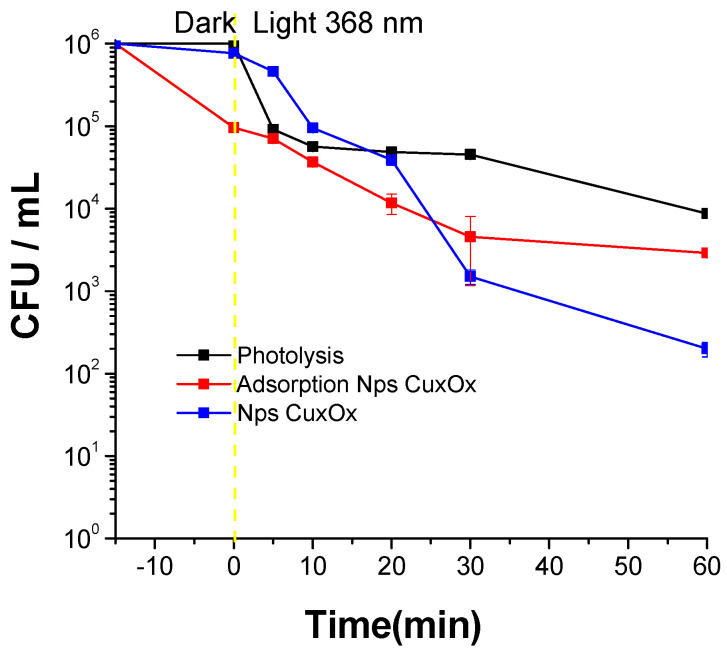
Disinfection kinetics of *E. coli* under different conditions. Control photolysis at 368 nm. Adsorption process for NPs Cu/Cu_2_O/CuO. Photocatalytic process for this material.

As seen in Figure 4, there are several behaviors of the bacteria under the considered system. Firstly, the photolysis control (black line in Figure 4) shows almost two logarithms of inhibition. As it is well known, UVA radiation has broadly used antimicrobial properties. Figure 4 also shows the adsorption control without light for Cu/Cu_2_O/CuO NPs at 50 mJ (red line), and finally, Cu/Cu_2_O/Cu NPs at 50 mJ using light at 368 nm (blue line). These decreases in adsorption control could be the product of a few interactions of NPs with polar heads of phospholipids in the membrane cell of bacteria. The NPs of small sizes (NPs < 40 nm) can migrate to interior cells, but others with larger sizes (NPps > 40 nm) can participate in the adsorption process. Finally, when observing the photoactivated system (blue line), there are some aspects to consider, including the main inhibition effect in NPs formed by laser ablation (black line) after 60 min of treatment with less than 10^4^ CFU.

### 2.4. Phase Transition Experiments by Infrared Spectroscopy

**Figure 5:**
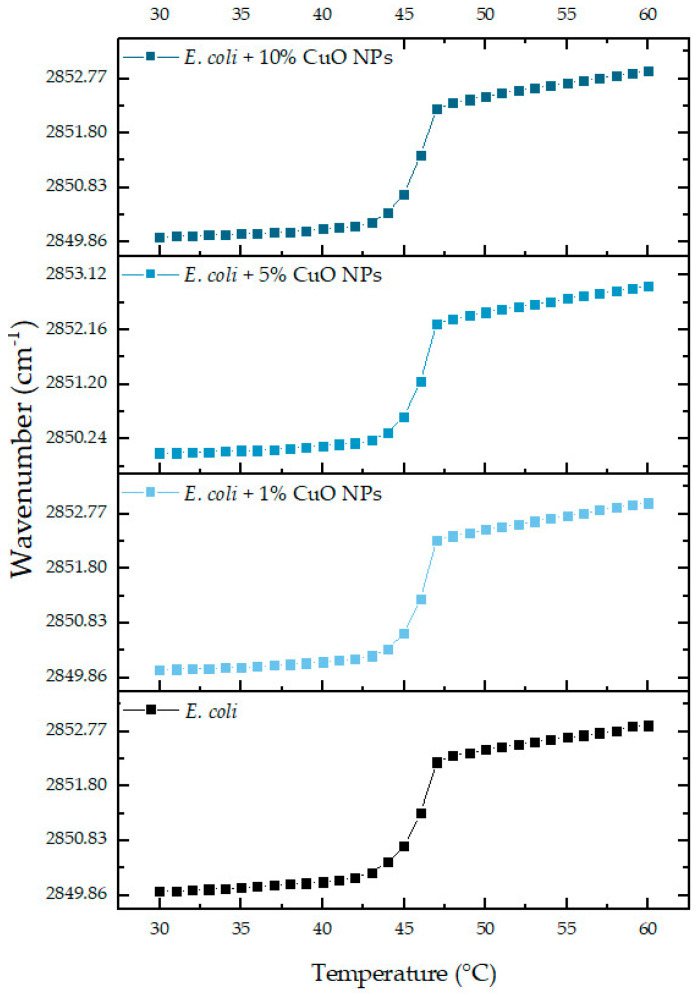
Peak positions of the νsCH_2_ vibration bands of the methylene groups as a function of the temperature of the representative model of *E. coli* (DMPE: DMPG: CL; 75:20:5) in buffer (10 mM HEPES, 500 mM NaCl, 1 mM EDTA, at pH 7.4).

One of the most studied physicochemical parameters to follow the interaction of exogenous molecules with membranes is the wavenumber peak position of the νs CH_2_ band. This vibration is related to the lipid order and packing of the hydrophobic core of membranes, and depending on temperature, the lipid bilayer has different states. At lower temperatures, in the gel phase, ν_s_CH_2_ lies around 2850 cm^−1^; while at higher temperatures, in the liquid crystalline phase, the band lies around 2853 cm^−1^. Following the phase transition, it is possible to calculate the main transition temperature (T_m_) of a lipid system. The results of the phase transition measurements of the multicomponent lipid system representative of the *E. coli* membrane are summarized in Figure 5.

**Figure 6:**
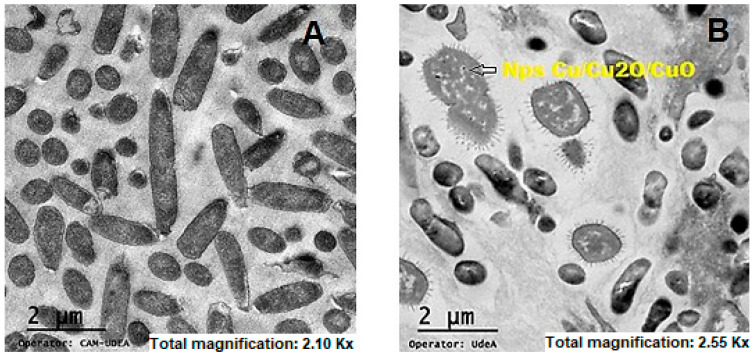
TEM micrograph of *E. coli*. (**A**) Control photolysis disinfection, (**B**) after photocatalytic exposition to Cu/Cu_2_O/CuO NPs.

To understand the chemistry effect on membrane microorganisms, TEM micrographs of *E. coli* were compared with and without photocatalytic treatment using the Cu/Cu_2_O/CuO NPs (Figure 6).

**Figure 7:**
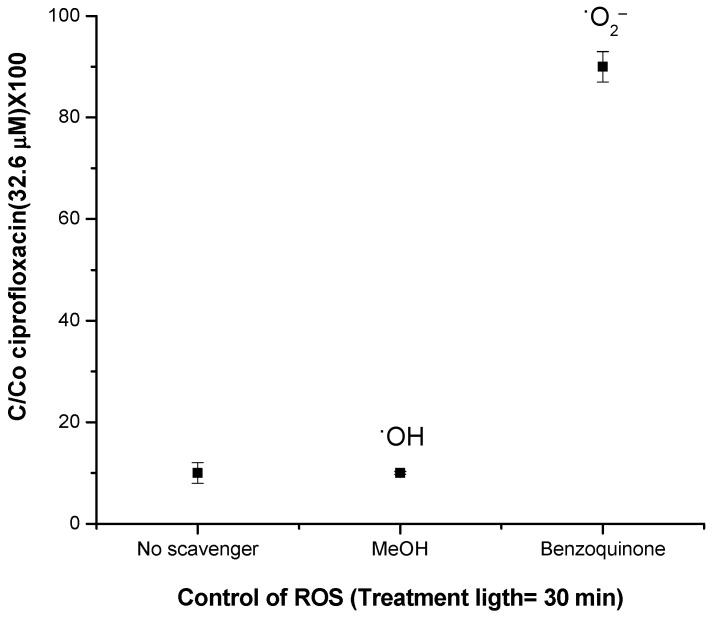
ROS estimation using the selective spin trap for hydroxyl and superoxide anion radicals for 30 min of treatment.

To suggest whether the release of these ROS is significant or if the mechanism is simply associated with the effect of light on modifying the hydrophilicity of the material, analyses were carried out with selective scavengers of superoxide anion radicals (benzoquinone) and methanol (hydroxyl). These tests were performed with a probe organic molecule (ciprofloxacin) sensitive to these ROS (Figure 7). It was not possible to perform these tests in situ with the bacteria because methanol is highly disinfectant and the benzoquinone molecule can interact through πι-stacking with aromatic molecules in the cell membrane, yielding inconclusive results.

## 3. Discussion

In Figure 1A, the spectra establish differences at 200–400 nm, which are characteristic bands of CuO and Cu_2_O absorption. The bands at 270 nm and 380 nm are assigned to CuO and Cu_2_O, respectively [19]. However, other authors indicate that the band near 210 nm corresponds to Cu_2_O and that at 300 nm corresponds to CuO [20]. The presence of Cu_2_O can be attributed to the generation of chemical reactions such as the dissociation of the oxide molecules in the aqueous medium, due to the pressure and temperature in the PLA plasma column. The band around 270 nm is attributed to induced charge transfers between the 2p orbitals of oxygen and Cu^2+^ ions from the 4s band, while the band around 590 nm corresponds to the inter-band transition of the copper electron from the upper level of the valence band [21]. The optical response of colloidal copper nanoparticles at ~600 nm is due to the transition between the upper-level copper electron band to the valence band, also known as Surface Plasmon Resonance (SPR) [22]. SPR results were reported with metallic nanoparticles and not from oxides [23]. Similarly, H. S. Desarkar and co-workers established the SPR of colloidal copper nanoparticles with a band at 550–640 nm, which coincides with the results obtained from UV-Vis spectra [24]. This confirms the formation of copper nanoparticles by the dissociation of molecules and nanoparticles from their oxides [25], generating metallic copper particles that react with the medium. On the other hand, it was found that the absorbance of the nanoparticles increases with ablation time, correlating to a higher number of nanoparticles in the colloidal solution [26]. NPs with mixed oxide and metallic copper (50 mJ) were selected for band gap determination and subsequent antimicrobial studies. In Figure 1B, the NPs are interesting in several aspects, in addition to the simplicity of the experiment (only a metal matrix, water, and a laser). This figure depicts that the most frequent NPs are less than 40 nm and are monodispersed and spherical shaped, which could allow them to then permeate the cell membrane of microorganisms. The distribution of the nanoparticles is homogeneous. Most of the particles are below 50 nm in size. However, there is a smaller quantity between 50 and 100 nm. Figure 1C shows surface analyses for NPs < 100 nm indicating that their surfaces are CuO (elemental analyses for Table 2), which suggests that this might be the most reactive part. This aspect is important because the structural configuration of the NPs contributes to their attack on bacteria. Since the majority are small nanoparticles, they can enter the microorganism, losing their surface layer and exposing Cu^1+^ to oxidative reactions in the intracellular environment.

As can be seen in Figure 1D, using Tauc’s method, the band gap for NPs obtained by laser ablation was 3.96 eV (Equation (1)). The band gap allows us to establish the region of the electromagnetic spectrum in which the material absorbs. This way, it can be irradiated with the specific lamp to achieve its best disinfectant catalytic efficiencies. In this context, to carry out the photoactivation process, UVA lamps with an emission centered at 368 nm were chosen.

In Figure 2, the thickness determination of CuO NPs on FTO by UV-Vis was calculated for a value of 685.3 nm, which allows the development of a prosperous electrochemical study [27].

Figure 3A shows the reduction of CuO to Cu_2_O at −0.74 V (Equation (1)), and the second peak, −0.95 V (Equation (2)), confirms the reduction of Cu_2_O to Cu at −1.31 V (Equation (3)). The reverse cycle evidences the oxidation of the deposited copper at a potential −0.29 V (Equation (3)). The reactions correlate with those found in the Pourbaix curves for copper (II). During the experiments, bubbling occurred on the surface of the FTO working electrode at −0.29 V, associated with copper deposition and a reduction in the acidic medium. The crossover (“nucleation loop”) of the electrocrystallization processes is also evident at −1.0 V [28]. Nucleation and growth became noticeable with the presence of a red color, which was more intense than the NPs obtained by LA.

2CuO + 2H^+^ + 2e^−^ → Cu_2_O + H_2_O      −0.74V (1)

Cu_2_O +2H^+^ + 2e^−^ → 2Cu + H_2_O         −1.31 V (2)

Cu → Cu^2+^ + 2e^−^      −0.29 V (3)

Figure 3B displays charges and discharges where the signal established a repeatability of 95%. The NPs CuO on FTO presents a low coloration time of 5.79 s while the relaxation time to resume transparency at positive potentials is 4.4 times longer, reaching 25.5 ± 2.3 s. Thus, cathodic processes with charge insertion are less impeded than oxidations, which require the release of adsorbed lithium ions on the surface of the deposited NPs. However, the coloring and bleaching processes are carried out in less than 40 s.

On the other hand, the contrast of the films remained at 43.8 ± 0.53%T, which exceeds the value requested in the manufacture of electrochromic devices of 40%T [29]. The loss of contrast in the film may be due to the detachment of the film on the FTO electrode or limitations in the ionic diffusion of lithium on the membrane, which is important in the electrochromic process, as shown in Equation (4) [30] and Figure 3C.

Cu_x_O + yLi^+^ + x e^~^  ↔  Li_y_Cu_x_O (4)

(transparent)          (brown)

The efficiencies are comparable with other studies obtained for WO_3_, which reported 41.6 and 19.8 cm^2^ C^−1^ [31] and poly(3-hexylthiophene-2,5-diyl)-copper oxide composites.

As shown in Figure 3D regarding the voltammogram carried out with and without light in the presence of peroxide, it was possible to estimate the values of the valence and conduction bands for the synthesized Cu/Cu_2_O/CuO. It was observed that the oxidation of Cu to Cu^2+^ is favored in the presence of light, demonstrating the semiconductor properties of the material. On the other hand, the presence of an oxidizing agent such as H_2_O_2_ shows reactivity towards Cu^1+^ and Cu^2+^ present in the mixed material, being more beneficial for the latter and possibly locating it on the periphery of the material. This is important because intracellularly, the microorganism presents metalloenzymes that oxidize/reduce H_2_O_2_. Conducting the assay in the presence of this agent suggests the possibility of Fenton-like reactions inside the bacterium, where Cu^1+^ reacts with H_2_O_2_ to generate hydroxyl radicals.

In the same context, three different interactions between the bacteria and the material are observed. The first is adsorption, where the zero-charge potential of the material can interact with the residual charge of the microorganism’s membrane, which is positive in the case of *E. coli*. This allows the nanoparticles to quickly move to the surface of the bacteria and, depending on their size, penetrate it and disrupt metabolic processes. The PZC potential of the material was measured, finding a value of +7 mV at pH 6.2, which would allow this interaction for nanoparticles larger than 50 nm (which are few according to the SEM histogram distribution), contributing to their mechanical destruction in 2 log units up to 30 min. On the other hand, the effect of photolysis is well known, as light can change the hydrophobicity of the surface of the material. This effect would better drive Van der Waals forces, electrostatic, hydrophobic, and receptor-ligand interactions, generating a similar effect to that mentioned earlier. However, all these changes contribute to allowing nanoparticles smaller than 50 nm to permeate the membrane and disrupt metabolic processes in the presence of oxidizing agents within the microorganism. The interaction of light is an external agent to the microorganism, which means that the photocatalytic activation of the material contributes to the predisposition of the membrane to the entry of smaller NPs. The physicochemical membrane study was conducted after 30 min of treatment outside the IR equipment container. This is because the IR sample holder is completely enclosed and cannot accommodate a light pumping system to follow the assay step by step. Nevertheless, it is conclusive that there are preliminary factors affecting membrane conditions that, while few are destroying it, facilitate the permeation of smaller NPs that seem to be responsible for an additional 2 log units in disinfection. In this context, to better understand the behavior of the material with and without light, cyclic voltammetry was used under the assay conditions without the microorganism. Notably, the assay was conducted in the presence of H_2_O_2_ to analyze the reactivity of Cu(I) in the material, which is susceptible to carrying out Fenton-like reactions. With and without light, Cu species are present, and in the presence of H_2_O_2_ and light, it rapidly oxidizes to Cu(II). Conditions that could favor the release of hydroxyl radicals in the presence of intracellular H_2_O_2_ are shown in Figure 3E [32,33,34,35].

Figure 4 suggests that the Cu/Cu_2_O/Cu NPs interact with light, and these can inhibit the production of Reactive Species (RS). Three significant reactive species can interact with cell molecules, Reactive Oxygen Species (ROS), Reactive Nitrogen Species (NOS), and Reactive Chloride Species (RCS), and each one is interesting and can be generated in different places of biological cells such as membranes or inside the cytosol. Among all ROS, O_2_^•−^ (superoxide anion radical) and hydrogen peroxide (H_2_O_2_) are considered the most highly produced ROS in the cells, and in the absence of any antioxidant, they can lead to cell death. CuO is widely known for its disinfection activity. The ROS generated during the photocatalytic process has strong oxidizing properties, leading to the breakdown of cell walls, membranes, and other vital cellular components of microorganisms. In regard to the previously realized discussion, Cu/Cu_2_O/Cu can produce radical species. However, more analysis is necessary to understand the formation, kinetic, and effect of these species.

As shown in Figure 5, the change in the peak position of the symmetric stretching vibration band of the methylene group, as a function of the temperature of the multi-component system, produced a sigmoidal curve, which reflects the degree of molecular aggregation of these systems [36]. The transitions of the *E. coli* system in the absence of NPs have a T_m_ of 49.5 °C. Increasing concentrations of the Cu/Cu_2_O/Cu NPs were not able to alter the cooperativity of the phase transition of the phospholipid system. Even at the highest concentration of the NPs evaluated (10 molar), there was no shift in T_m_. The results suggested that the Cu/Cu_2_O/Cu NPs did not exert their effect through an interaction with the phospholipids present in the bacterial membrane. It is highly probable that according to the size (less than 50 nm) and z potential of NPs (7.5 mV), understanding the last one as a measure of the magnitude of electrostatic (or charge) repulsion or attraction through the potential of the double layer (diffuse and stern), NPs have very low interaction with polar heads of phospholipids and easily penetrate the hydrophobic core without disturbing the hydration or the bilayer structure of the membrane. For this reason, there was no change in the frequency of infrared absorption of the CH_2_ groups, reaching the interior of cytosol and acting as disinfectant material inside [37].

As can be seen, Figure 6 shows, on the one hand, entire bacteria that are deeper and were not reached by the ultra-microtome blade, and there are individuals and others fixed right in the division process, and on the other hand, bacteria cut through the middle, in which the integrity of the membrane can be observed by the interaction models previously shown, as well as the presence of NPs and some metal oxide aggregates inside the cytosol, which are consistent with all the results previously analyzed. A bacterium cultured under the same experimental conditions, without treatment with nanoparticles and only activated by interaction with the Xenon lamp, has been isolated. This shows that the applied lamp intensity is insufficient to cause damage to the microorganism, and it is precisely the migration of the particle through the membrane that exposes the bacterium to structural damage. In this context, the following figure includes the TEM image using light as a photolysis control in the disinfection process.

Figure 7 depicts that in the absence of scavengers, 90% of the probe compound was eliminated. In addition, in the presence of methanol, which can trap hydroxyl radical species, the elimination of the pollutant model remained unaffected, which highlights the absence of this radical under work conditions. Interestingly, the experiment with benzoquinone showed strong inhibition of the process. Only 10% of the probe compound was eliminated in 30 min. Therefore, the results suggest that the superoxide anion radical is the main species generated in the photocatalytic process.

## 4. Materials and Methods

### 4.1. Materials

Copper chloride (CuCl_2_), Fluorine-doped tin oxide electrodes (FTO), Lithium perchlorate (LiClO_4_), acid perchloric (HClO_4_), sodium hydroxide (NaOH), reference electrode silver/silver chloride (Ag|AgCl|KCl (sat)), and Plate Count Agar (PCA) all were purchased from Sigma-Aldrich (Middlesex County, Massachusetts, USA) and used without further purification. For the simulated membrane, 1,2-dimyristoyl-sn-glycero-3-phosphoethanolamine (DMPE, Lot. 140PE-63), 1,2-dimyristoyl-sn-glycero-3-phosphoglycerol sodium salt (DMPG, Lot. 140PG-167), and 1′,3′-bis [1,2-dimyristoleoyl-sn-glycero-3-phospho]-glycerol sodium salt (CL, Lot. 750332P 200MG-A-030) were purchased from Avanti Polar Lipids (Birmingham, AL, USA), and 2,2’,2″,2‴-(Ethane-1,2-diyldinitrilo) tetra acetic acid (C10H16N2O8, EDTA) was from Amresco (Medellin, Colombia). Water HPLC was obtained from thermo-scientific smart2pure3 equipment (Waltham, Middlesex County, Massachusetts, USA). Supported lipid bilayers (SLBs) were prepared in situ in a BioATR II cell.

### 4.2. Synthesis of Colloidal Cu/Cu_2_O/CuO

**Figure 8:**
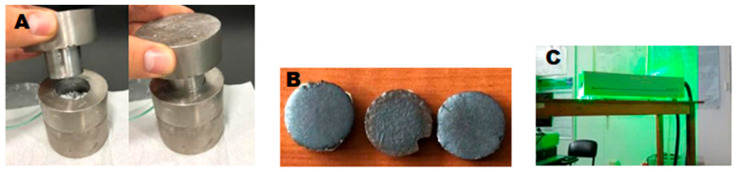
(**A**) Cell for sintered, (**B**) Target Cu/Cu_2_O/CuO, (**C**). Laser 1064 nm.

- Colloidal NPs were synthesized according to Corrales [15] using the conventional technique of pulsed laser ablation. As the first step, a solid quantity of CuO (used without further purification) was pressed under 2 tons in a mechanical press and heated for 12 h at different rates (0–100 °C in 2 h, 100–300 °C in 2 h, 300–500 °C in 3 h, and 500–800 °C in 5 h) until a sintered material was obtained.
- This solid matrix tablet was submerged in water and exposed to the impact of a 1064 nm Nd:YAG (Nd: Y_3_Al_6_O_12_) laser at energy levels of 25, 50, and 80 mJ for 5 and 10 min of contact. Each material was synthesized independently, as shown in Figure 8A–C.

### 4.3. Evaluation of Electronic and Electrochemical Properties for Cu/Cu_2_O/CuO on FTO

The deposition of nanomaterials was performed by dip coating on previously washed FTO (SnO_2_:F) 15 Ω/sq on a glass substrate (XOP Glass, Castellón—Spain), and cut with approximate areas of 25 mm × 25 mm. Thus, 20 immersions were performed for 1 min in colloidal Cu/Cu_2_O/CuO NPs with 15 s of drying outside at room temperature. After finishing the deposition cycle, they were sintered at 500 °C for 1 h in an oxygen atmosphere. The band gaps of nanomaterials were obtained by the Tauc method (Equation (5)), using some variables from UV-VIS spectra.

(α*hv*) = α(*hv* − *Eg*) 1 *r* (5)

where *r* = ½ for allowed direct transitions, 3/2 for forbidden direct transitions, 2 for allowed indirect transitions, and 3 for forbidden direct transitions, *h* is Planck’s constant, *v* is the frequency of photons, α the absorption coefficient, α is a proportionality constant, and *Eg* the energy gap transitions [15].

The electrochemical properties of the NPs wWere determined by cyclic voltammetry between −1.5 and 1.5 V in a single-compartment cell consisting of an FTO working electrode, naked and doped (with NPs), Ag/AgCl (sat) as the reference electrode, and platinum wire as an auxiliary electrode in LiClO_4_ at 0.1 mol L^−1^ and 50 mVs^−1^. The electrochromic response of the deposited films was determined by applying charges and discharges of potentials between 1.5 and −1.5 V vs. Ag/AgCl and the detection of the transmittance at 580 nm. The average charge density was 4.06 ± 0.4 mC on electrodes with an area of 0.60 ± 0.01 cm^2^.

### 4.4. Evaluation of Properties as Disinfectant Material Cu/Cu_2_O/CuO against E. coli 25922

- To evaluate the antibacterial activity of colloidal NPs, photocatalytic processes were carried out, and their effect on bacteria was tested at different times. For this purpose, 14.5 mL of *E. coli* ATCC 25922 was placed in autoclave water at an optical density of 0.1 at 580 nm, corresponding to the exponential growth phase (EP) of microorganisms and 106 colony-forming units (CFU) mL^−1^, and was mixed with 0.5 mL of 50 mg/L colloidal NPs.
- This system was located under radiation to photoactivate the NPs, and after a certain time (0, 3, 10, 20, 30, and 50 min), the system was inoculated in Plate count agar. This procedure was also performed to serial dilutions of 1/10 systems in sterilized saline solution to obtain 105 and 104 CFU. Finally, the counting of colonies was performed after 24 h of incubation at 37 °C. For adsorption analysis, the systems (10^6^, 10^5^, or 10^4^ CFU) were seeded 15 min before photoactivation (the absence of light).
- For supported lipid bilayers, the simulated membrane of *E. coli* was composed of the DMPE:DMPG:Cl (75:20:5) lipid system. The infrared study was performed as follows: First, the background was taken using 20 mmol L^−1^ HEPES buffer, 500 mmol L^−1^ NaCl, and 1 mmol L^−1^ EDTA in the same temperature range. Subsequently, to coat the silicon crystal, a stock solution in chloroform of DMPE:DMPG:CL (75:20:5) was deposited [36]. The cell was filled with 20 µL of this lipid stock solution, and its solvent was evaporated, resulting in a lipid multilayer film. For in situ measurements, the cell was filled with 20 µL of buffer or Cu/Cu_2_O/CuO NPs in buffer and incubated at the phase transition temperature for 10 min. To determine the position of the vibrational band in the range of the second derivative of the spectra, all the absorbance spectra were cut in the 2970–2820 cm^−1^ range and shifted to a zero baseline, and the peak picking function was included in Opus 8.8.4 Software. The results were plotted as a function of the temperature. To determine the transition temperature (T_m_) of the lipids, the curve was fitted according to the Boltzmann model to calculate the inflection point using Origin Pro 2018 of the thermal transition curves obtained.

### 4.5. Analytical Measurements

For UV-VIS spectra, the Perkin Elmer Lambda 9UV/VIS/NIR (Waltham, MA, USA) spectrometer was used. For size and morphology, the scanning electron microscope (SEM) JEOL JSM 6490 LV (Tokyo, Japan) was used. The cyclic voltammetry for oxidation states of species was performed in Autolab Potentiostat /galvanostat (Herisau, Switzerland). The homemade reactor for photocatalysis consisted of an aluminum box equipped with five UVA lamps (F8T/BLB, 15 W each, with an emission band at 368 nm). The SLBs in the BioATR II cell unit were integrated with a Tensor II spectrometer (Bruker Optics, Ettlingen, Germany) with a liquid nitrogen MCT detector using a spectral resolution of 4 cm^−1^ and 120 scans per spectrum. The desired temperature for the phase transition was set by a Huber Ministat 125 computer-controlled circulating water bath (Huber, Offenburg, Germany) with an accuracy of ±0.1 °C.

CIP (30.6 μM) evolution was followed using a UHPLC Thermo Scientific Dionex UltiMate 3000 Shirley, NY 11967, instrument equipped with an Acclaim™ 120 RP C18 column (5 μm, 4.6 × 150 mm) and a DAD detector. A mixture of formic acid (10 mM, pH 3.0) and acetonitrile was used as the mobile phase, and the CIP detection was set at 278 nm. For scavengers, we used benzoquinone and methanol one hundred times more concentrated than the drug to ensure the radical is scavenged.

## 5. Conclusions

The Cu/Cu_2_O/CuO NPs seem to reach the bacteria without damaging the phospholipid bilayer (according to simulated membrane, z potential, and TEM images) and easily cross this barrier to the cytosol, and there all the biological damage is generated by reactive species, causing a disinfection effect on *E. coli* and a considerable decrease in the CFU. The combined Cu, CuO, and Cu_2_O phases with their different bandgaps may be responsible for the improved photodegradation due to the increased charge separation efficiency and an extended range of photoexcitation while decreasing the recombination of photogenerated electrons and holes. More studies using spin traps for ROS in situ are necessary to understand the disinfection mechanism. The electrochromic results of Cu/Cu_2_O/CuO Ns make them potential coating materials to be used as indicators in the presence of microorganisms that present acid reductions in the adhered substrates. This article is a resource for further studies that require a protocol for the disinfection and synthesis of nanoparticles with mixed oxides using laser ablation.

## Figures and Tables

**Table 1:** Cu/CuO/Cu_2_O materials developed from synthesis and applications.

Material	Mechanism	Synthesis Procedure	Application
Cu/CuO/Cu_2_O heterojunction nanodisks [9].	Photocatalyst generating of ^•^O_2_^−^	Solvothermal method	Degradation of Rhodamine dye.
Cu/CuO/Cu_2_O NPs [10].	Reactive oxygen species (ROS)	Chemical synthesis	Cytotoxic activity against breast cancer cell lines (MCF-7).
Cu/CuO/Cu_2_O [11].	No specific mechanism	Chemical synthesis	p-nitroaniline degradation
Cu/CuO/Cu_2_O nanoplate polymer nanocomposites, [12].	No specific mechanism	Chemical synthesis	Optoelectronic device application
Cu/CuO/Cu_2_O Nanocrystals within Hybrid Nanofibers [13].	No specific mechanism	A reduction temperature of up to 800 °C in 15% H_2_ balanced in N_2_	Arsenic(V) removal
Cu/CuO/Cu_2_O Thin Film [14].	No specific mechanism	Thin-film deposition method	No application was reported.

**Table 2:** Elemental composition of NPs Cu/Cu_2_O/CuO corresponding with CuO on the surface.

Element	Atomic %
O	35.32
Cu	64.38

**Table 3:** Electrochromic results of Cu/Cu_2_O/CuO NPs deposited in FTO.

Nanoparticle Film	Bleaching Time (tb) (s)	Coloring Time (tc) (s)	Contrast ΔT	Optical Density ΔOD	Efficiency (η) (cm^2^/C)
CuO 50 mJ	25.5 ± 2.3	5.75 ± 0.9	43.8 ± 0.53	0.244 ± 0.052	36.0 ± 0.05

Average charge 4.06 ± 0.4 mC, average area 0.60 ± 0.01 cm^2^. Values are taken for 83 consecutive cycles with 95% repeatability between cycles.

## Data Availability

No new data were created or analyzed in this study. Data sharing is not applicable to this article.
